# Complying to the standard of integrated diabetic care

**Published:** 2012-09-04

**Authors:** C.A.J Ketelaars, H.L.G Scheijven

**Affiliations:** Health Care Inspectorate, The Netherlands; Health Care Inspectorate, The Netherlands

**Keywords:** integrated care, compliance, supervision

## Abstract

**Introduction:**

In 2008, the Dutch Ministry of Health introduced policy reforms to enable integrated care for chronic diseases in combination with an integrated payment system. Implementation of these integrated care programs for diabetes, vascular risk management and COPD, by so-called integrated care groups, has started throughout the country.

**Purpose:**

The Dutch Health Care Inspectorate (DHI) is the regulator for integrate care. Supervision is carried out on the quality of care delivered by care groups, regarding the standard of diabetic care.

**Methods:**

In 2011, 20 care groups are at random selected and each care group is visited by two health care inspectors. Quality of care is assessed on topics, such as: multidisciplinary patient files, personal care plans, education/prevention measures, self management and transparency of quality. Information is obtained by interviewing general practitioners, practice nurses and the management of the care group. Furthermore policy documents and patient files are studied.

**Results and conclusion:**

Preliminary results show that care groups don’t (want to) take full responsibility for the quality of integrated diabetic care they provide. Final results will be presented at the conference.

Powerpoint presentation available at http://www.integratedcare.org at congresses – San Marino – programme.

